# Automated Synthesis of Fluorine-18 Labeled CXCR4 Ligand via the Conjugation with Nicotinic Acid *N*-Hydroxysuccinimide Ester (6-[^18^F]SFPy)

**DOI:** 10.3390/molecules25173924

**Published:** 2020-08-27

**Authors:** Falguni Basuli, Xiang Zhang, Tim E. Phelps, Elaine M. Jagoda, Peter L. Choyke, Rolf E. Swenson

**Affiliations:** 1 Chemistry and Synthesis Center, National Heart, Lung, and Blood Institute, National Institutes of Health, Rockville, MD 20892, USA; xiang.zhang2@nih.gov (X.Z.); rolf.swenson@nih.gov (R.E.S.); 2 Molecular Imaging Program, National Cancer Institute, National Institutes of Health, Bethesda, MD 20892, USA; tim.phelps@nih.gov (T.E.P.); ejagoda@mail.nih.gov (E.M.J.); pchoyke@mail.nih.gov (P.L.C.)

**Keywords:** fluorine-18, fluorination on the Sep-Pak, SFPy, CXCR4, T140

## Abstract

The C-X-C motif chemokine receptor 4 (CXCR4) is a seven-transmembrane G protein-coupled receptor that is overexpressed in numerous diseases, particularly in various cancers and is a powerful chemokine, attracting cells to the bone marrow niche. Therefore, CXCR4 is an attractive target for imaging and therapeutic purposes. The goal of this study is to develop an efficient, reproducible, and straightforward method to prepare a fluorine-18 labeled CXCR4 ligand. 6-[^18^F]Fluoronicotinic acid-2,3,5,6-tetrafluorophenyl ester (6-[^18^F]FPy-TFP) and nicotinic acid N-hydroxysuccinimide ester (6-[^18^F]SFPy) have been prepared using ‘fluorination on the Sep-Pak’ method. Conjugation of 6-[^18^F]SFPy or 6-[^18^F]FPy-TFP with the alpha-amino group at the N terminus of the protected T140 precursor followed by deprotection, yielded the final product 6-[^18^F]FPy-T140. The overall radiochemical yields were 6–17% (*n* = 15, decay-corrected) in a 90-min radiolabeling time with a radiochemical purity >99%. 6-[^18^F]FPy-T140 exhibited high specific binding and nanomolar affinity for CXCR4 in vitro, indicating that the biological activity of the peptide was preserved. For the first time, [^18^F]SFPy has been prepared using ‘fluorination on the Sep-Pak’ method that allows rapid automated synthesis of 6-[^18^F]FPy-T140. In addition to increased synthetic efficiency, this construct binds with CXCR4 in high affinity and may have potential as an in vivo positron emission tomography (PET) imaging agent. This radiosynthesis method should encourage wider use of this PET agent to quantify CXCR4 in both research and clinical settings.

## 1. Introduction

Chemokine receptors (CRs) are seven-transmembrane G protein-coupled receptors that trigger intracellular signaling and drive cell polarization, adhesion, and migration [1]. The chemokine receptor type 4 (CXCR4) is a distinctive member of the CR family, possessing additional functions during embryonic development, and has been widely studied with the initial discovery that CXCR4 is one of the co-receptors for human immunodeficiency virus (HIV) entry into target cells [2,3,4,5]. CXCR4 is expressed on most hematopoietic cell types and stem cells. The structure of CXCR4 consists of 352 amino acid residues comprising an N-terminal domain, seven TM domains, three extracellular loops (ECL), three intracellular loops (ICL), and a C-terminal domain [6]. This chemokine receptor is overexpressed in a variety of cancers including kidney, lung, brain, prostate, breast, pancreas, ovarian, and melanomas [7,8,9]. The natural ligand for CXCR4, CXCL12, also known as a stromal cell-derived factor (SDF)-1, is a 67-residue large peptide that is expressed and secreted in different organs such as the liver, lung, kidney, brain, and bone marrow [3,4,10,11]. The CXCL12/CXCR4 axis plays an important role in normal cell migration, inflammation, and immune surveillance. In addition, this axis is required for embryonic development and certain physiological functions including hematopoiesis, organogenesis, and vascularization [12]. Dysregulation of CXCL12/CXCR4 signaling is associated with numerous pathological conditions, including various types of cancers, chronic inflammatory diseases, cardiovascular diseases, and immunodeficiencies [13,14,15]. Therefore, CXCR4 is an attractive target for imaging cancerous lesions and their microenvironment which may have clinical applications towards diagnosis and patient selection for not only targeted therapeutics but immunotherapies as well [16,17,18,19,20].

**Figure 1:**
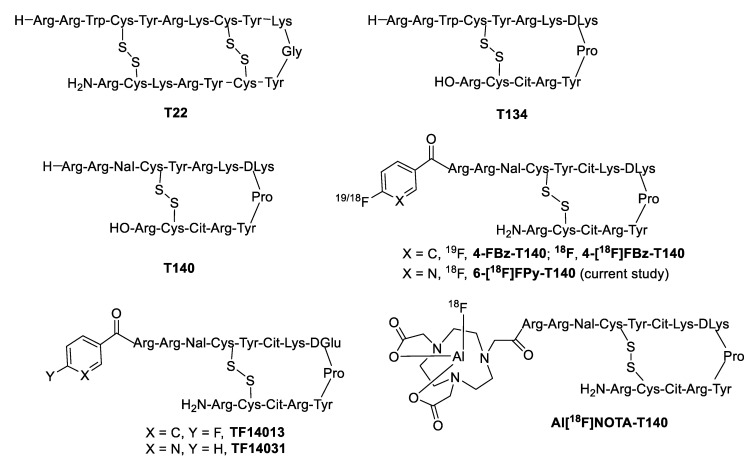
Structures of C-X-C motif chemokine receptor 4 (CXCR4) ligands.

Numerous truncated peptide analogs that mimic the function of CXCL12 have been reported [17,21]. Tamamura et al. described the synthesis of novel 14-amino acid peptide inhibitors (T134, T140) based on the structure of their previously reported 18-amino acid peptide, T22, for inhibitory activity against HIV-1 entry [22,23]. These peptides demonstrated high anti-HIV activity with significantly less cytotoxicity when compared to T22. Inhibitory activity has also been detected against a variety of cancer types causing a reduction in metastasis and progression [24,25,26,27,28,29]. The same group observed enhancement of anti-HIV activity by introducing a 4-fluorobenzoyl group on a T140-based pharmacophore and developed 4-fluorobenzoyl-TN14003 (4-FBz-T140, Figure 1) [30]. The fluorine atom of 4-FBz-T140 could be replaced by fluorine-18 with no change in the structure of the peptide, thereby providing a potential candidate for a CXCR4 PET imaging agent.

In 2010, Jacobson et al. reported the synthesis of fluorine-18 labeled analog, 4-[^18^F]fluorobenzoyl-TN14003 (4-[^18^F]FBz-T140), in four steps using [^18^F]SFB from which CXCR4 positive tumors were visualized in a xenograft mouse model at high tumor-to-background ratios [31]. These high tumor-to-background ratios were achieved by blocking the elevated binding of 4-[^18^F]FBz-T140 to red blood cells (RBCs) with non-radioactive standard 4-FBz-T140 peptide. However, due to the complex and time-consuming radiolabeling procedure, they developed another analog, (Al[^18^F]NOTA-T140), using the Al-F method [32]. Although the radiolabeling method was significantly simplified with high radiochemical yield and molar activity, the tracer showed high uptake in the liver and kidneys. Tamamura et al. developed a pyridyl analog (TF14031) of TF14013, which showed similar anti-HIV activity as that of TF14013 [30]. Therefore, we anticipated that the pyridyl analog of 4-[^18^F]FBz-T140 would retain the potency of the tracer while facilitating an easier and more efficient radiosynthesis method. Herein, the synthesis of [^18^F]SFPy, a pyridyl analog of [^18^F]SFB, using the ‘fluorination on the Sep-Pak’ method and subsequent conjugation of [^18^F]SFPy with the peptide precursor to produce the novel PET tracer, 6-[^18^F]FPy-T140, is reported. The simplicity of this labeling procedure allows us to successfully develop an automated synthesis method.

## 2. Result and Discussion

### 2.1. Chemistry and Radiochemistry

**Scheme 1:**
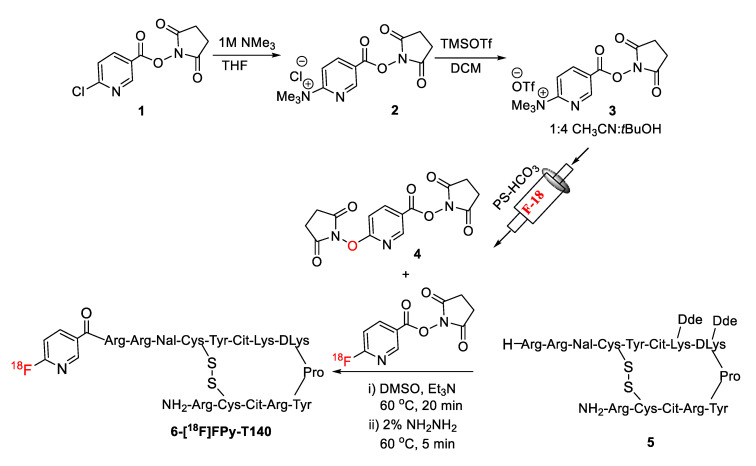
Preparation of [^18^F]SFPy by Sep-Pak fluorination method and conjugation with T140.

In a patent application, Siebeneicher et al. first reported the synthesis of 6-[^18^F]SFPy in a three-step reaction from fluorination of ethyl 6-chloronicotinate followed by hydrolysis and esterification [33]. Recently, Richard et al. reported a one-step synthesis of 6-[^18^F]SFPy by reacting ammonium precursors of NHS ester with [^18^F]KF/K_2_CO_3_/K_222_ either at room temperature or at 40 °C [34]. However, in this report, the low radiochemical yields for NHS and TFP esters were due to the instability of these esters under the radiolabeling conditions. Our recently developed ‘fluorination on the Sep-Pak’ method, which does not require elevated temperatures or the addition of a base, was therefore used to prepare 6-[^18^F]SFPy [35,36]. The precursor was prepared in three steps starting with commercially available 6-chloro nicotinic acid. The formation of NHS ester (**1**, Scheme 1) was achieved according to the literature method [37]. The substitution of chloride (**1**) with trimethylamine gas to prepare trimethylammonium salt (**2**) was unsuccessful, as reported [34,38]. Alternatively, compound **2** was successfully prepared in high yield (~80%) by using 1M trimethylamine solution in THF. Finally, anion exchange with trimethylsilyl triflate (TMSOTf) produced the desired triflate salt of the trimethylammonium precursor (**3**, Scheme 1).

**Figure 2:**
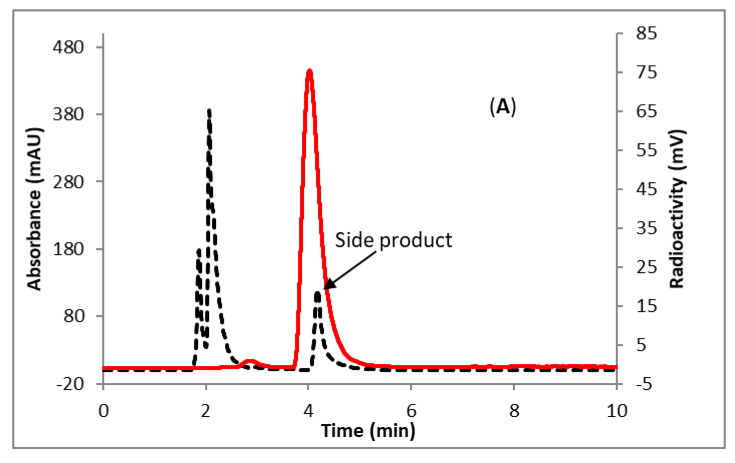

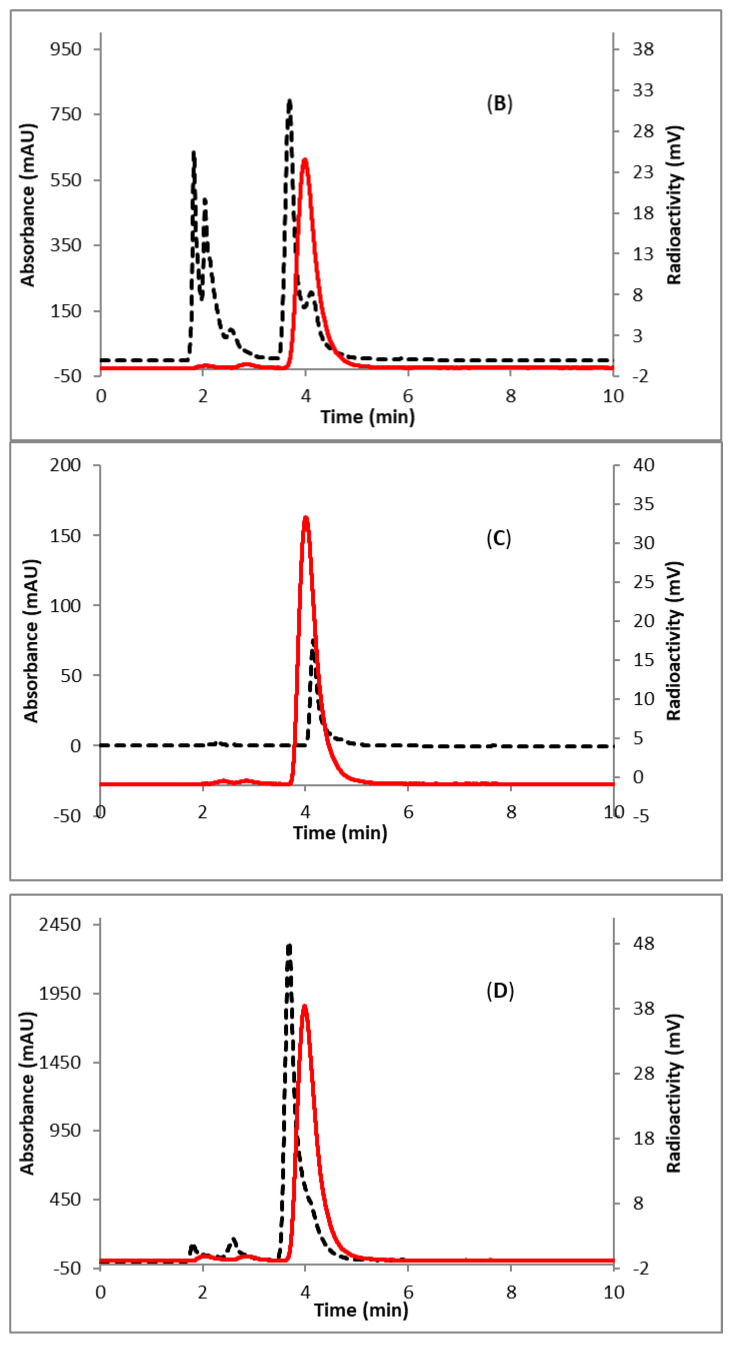

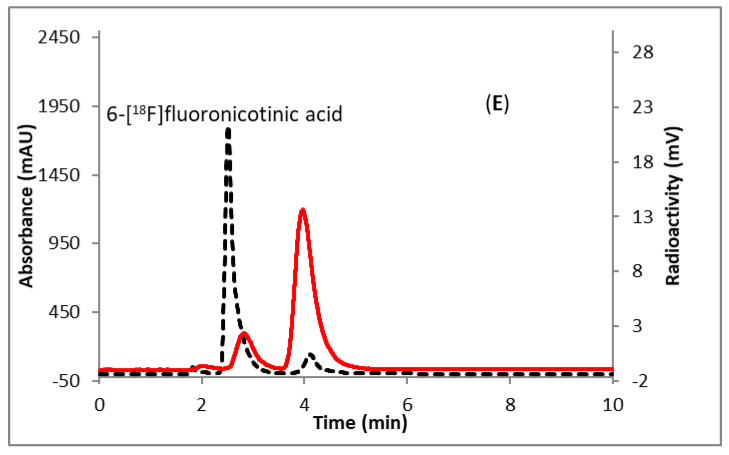
HPLC analysis (method B) of 6-[^18^F]SFPy (**A**) Crude reaction mixture; (**B**) Crude reaction mixture co-injected with the non-radioactive standard; (**C**) Pure; (**D**) Pure co-injected with the non-radioactive standard; (**E**) After 4h of labeling at room temperature co-injected with the non-radioactive standard, 6-fluoronicotinic acid. Solid line, in-line radio detector; dotted line, UV detector at 254 nm. Retention time (min): 6-[^18^F]SFPy, 4.0; compound **4**, 4.1; 6-[^18^F]fluoronicotinic acid, 2.8.

To test the efficacy of the ‘fluorination on the Sep-Pak’ method, 10 mg of precursor (**3**) in 500 uL of 1:4 acetonitrile, *t*-butanol solution was passed through the cartridge (PS-HCO_3_) containing fluorine-18 followed by flushing the cartridge with 1 mL acetonitrile. Over 80% of the fluorine-18 was eluted from the Sep-Pak. HPLC analysis of the reaction mixture revealed clean radiochemical conversion (Figure 2A). The identity of 6-[^18^F]SFPy was confirmed by comparing the HPLC retention time with co-injected, authentic nonradioactive standards (Figure 2B). The [^18^F]fluoride elution efficiency is comparable to the elution efficiency previously observed for the TFP-ester [35,36]. Reducing the amount of precursor (**3**) resulted in a lower elution of fluoride from the Sep-Pak. The analysis of the decayed reaction mixture of 6-[^18^F]SFPy by mass spectrometry revealed the formation of a known side product, NHS ester with succinimidyl ether linkage at the 6-position of the pyridine ring (**4**) [34]. The unreacted precursor from the crude reaction mixture of 6-[^18^F]SFPy was completely removed by passing the mixture through an activated oasis MCX plus cartridge (Figure 2C). However, the side product (**4**), which is potentially an active substrate for the conjugation reaction, was not removed by this purification method (Figure 2C). The identity of the pure 6-[^18^F]SFPy was confirmed by comparing the HPLC retention time with co-injected, authentic nonradioactive standards (Figure 2D). Slow hydrolysis of 6-[^18^F]SFPy to 6-[^18^F]fluoronicotinic acid was observed with time (15% conversion after 4h post radiolabeling) at room temperature (Figure 2E). Compound **4** was independently prepared by reacting compound **3** with N-hydroxysuccinimide (NHS) to quantify the concentration of this compound in the final solution of 6-[^18^F]SFPy. A calibration curve was generated using a known amount of **4** and using this curve a typical radiolabeling reaction starting with 8.9 mg of precursor (**3**) and 3.88 GBq (105 mCi) of [^18^F]fluoride, generated 46 µg (0.14 µmol) of side product, which is 7–11 mol% of the protected peptide precursor (**5**) we intended to use (3–5 mg, 1.26–2.11 µmol) for the conjugation reaction. The impurity (**4**) can be removed by a time-consuming HPLC purification followed by a solvent exchange process with significant loss of radioactivity due to transfer and decay of 6-[^18^F]SFPy. This whole purification process might not be advantageous over the presence of the impurity in the labeled prosthetic group. Moreover, the final radiolabeled peptide (6-[^18^F]FPy-T140), will be purified by HPLC which will remove the impurities generated by **4**. Therefore, no attempts were made to further purify the prosthetic group, 6-[^18^F]SFPy.

The solvent from the 6-[^18^F]SFPy solution was removed under vacuum and N_2_ flow at 45 °C with no significant loss of activity. Amide bond formation with protected peptide precursor (**5**, Scheme 1) in dimethyl sulfoxide was tested at room temperature, 40 °C, and 60 °C. The radiochemical conversion determined by analytical HPLC was 3%, 6%, and 33% respectively. The effect of further increasing temperature on the radiochemical conversion was not tested due to the presence of a disulfide bond that might not be stable at high temperatures. Finally, deprotecting the Dde protecting group of the labeled peptide with hydrazine solution followed by HPLC purification produced >99% radiochemically pure 6-[^18^F]FPy-T140.

After manual optimization of the radiolabeling procedure with a low amount of radioactivity, we focused on the development of the automated synthesis of 6-[^18^F]FPy-T140 on a GE FX-N Pro Module. The direct radiolabeling of biomolecules such as peptides or proteins is difficult due to the usual requirement of high temperature and base to form the C-^18^F bond. Although there are a few examples of direct fluorine-18 labeling of peptides, labeling is typically performed using an indirect method [39,40,41,42]. Therefore, a fully automated synthesis is challenging due to the complexity of the indirect labeling method [43,44,45,46,47]. The steps involved for the preparation of fluorine-18 labeled prosthetic group via currently developed Sep-Pak method (catching of fluorine-18 on an anion exchange cartridge, drying of the cartridge, release of fluorine-18 with precursor), are equivalent to the initial processing of fluorine-18 for conventional nucleophilic fluorination (catching of fluorine-18 on an anion exchange cartridge, the release of fluorine-18 with base, azeotropic drying with acetonitrile). However, this method has several advantages including less time-consuming with no added base, resulting in producing the radiolabeling synthon in a significantly simplified process and comparable yield to the conventional labeling method. The simplified process of the ‘fluorination on the Sep-Pak’ method, combined with minor modifications on the GE module, readily allows for automated indirect labeling of the peptide.

**Figure 3:**
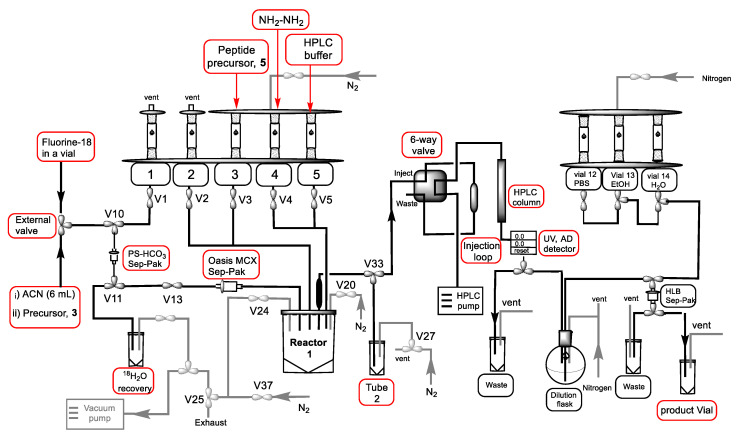
Schematic diagram of the automated synthesis of 6-[^18^F]FPy-CONH-T140 on GE FX-N Pro Module.

**Figure 4:**
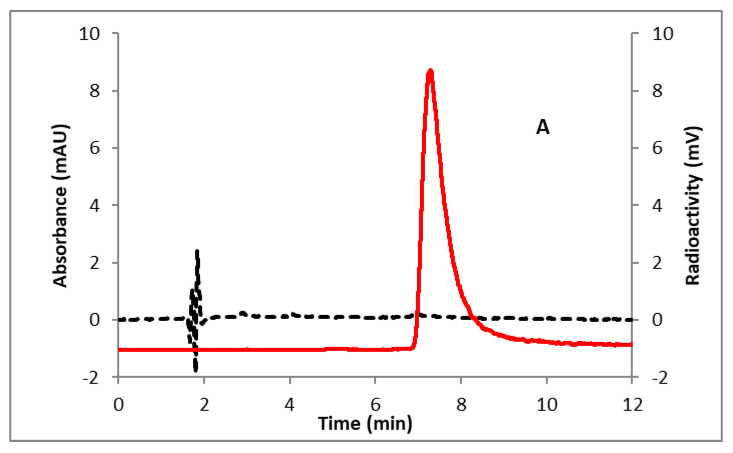

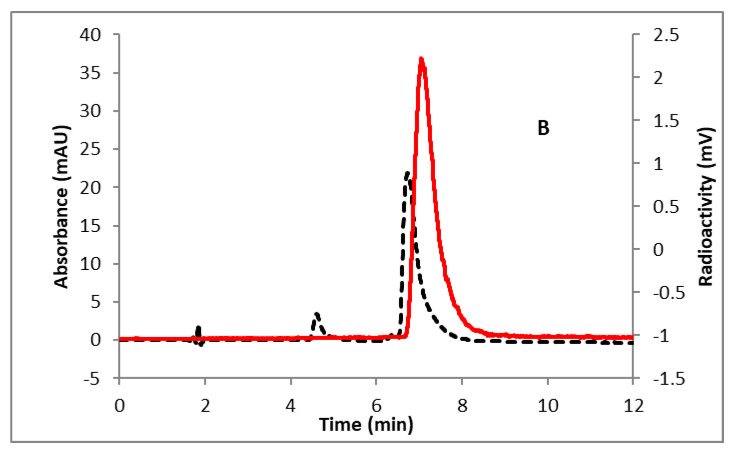
HPLC analysis (method B) of (**A**) 6-[^18^F]FPy-T140; (**B**) 6-[^18^F]FPy-T140 co-injected with the non-radioactive standard. Solid line, in-line radio detector; dotted line, UV detector at 254 nm. Retention time (min): 6-[^18^F]FPy-T140, 7.3.

An external three-way valve was added before the V10 valve (Figure 3) on the GE Tracerlab module (GE FX-N Pro) to accomplish the Sep-Pak preparation of 6-[^18^F]SFPy. An Oasis MCX cartridge was incorporated between V13 and reactor 1 for the purification of 6-[^18^F]SFPy esters. The entire process was automated except the step to pass the precursor solution through the Sep-Pak containing fluorine-18. This part was done manually for better control of the elution rate. To perform this operation a line from the external valve was kept out of the hot cell while the rest of the system was inside the hot cell. The peptide precursor (3–5 mg) in DMSO, 2% (*v*/*v*) hydrazine, and HPLC buffer were added in Vials 3–5, respectively (Figure 3). Phosphate-buffered saline (PBS) (pH 7.4), ethanol, and water were added in Vials 12–14, respectively for the final formulation of 6-[^18^F]FPy-T140. The overall radiochemical yield (2 steps) of the synthesis was 6–17% (*n* = 15, decay corrected) in a 90 min procedure. The radiochemical purity was >99% (Figure 4A) with a molar activity of 32–100 GBq/µmol. The comparable molar activity with other routinely prepared fluorine-18 labeled tracers in our lab and the absence of any UV peak (Figure 4A) indicated that all chemical impurities were successfully removed. The identity of the product was confirmed by comparing the HPLC retention time with co-injected, authentic nonradioactive standards (Figure 4B). In a typical reaction starting with 7.3 GBq (197 mCi) of [^18^F]fluoride on the Sep-Pak, 0.7 GBq (19 mCi) of the product was obtained. Fluorine-18 labeled nicotinic acid tetrafluorophenyl ester (6-[^18^F]FPy-TFP) was prepared according to the literature method to compare the overall RCYs of 6-[^18^F]FPy-T140 prepared either using 6-[^18^F]SFPy or 6-[^18^F]FPy-TFP [35,36]. The conjugation of 6-[^18^F]FPy-TFP and deprotection of the Dde protecting group were performed following the same protocol used to prepare 6-[^18^F]FPy-T140 from 6-[^18^F]SFPy. Similar RCYs of [^18^F]FPy-T140 were observed from both the prosthetic groups (5–16%, *n* = 6 vs. 6–17%, *n* = 15).

### 2.2. In Vitro Binding Assays

**Figure 5:**
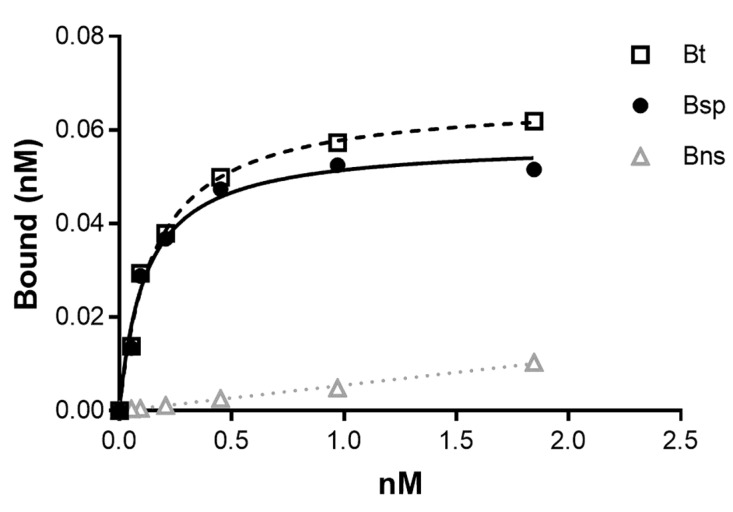
Representative plot from an in vitro 6-[^18^F]FPy-T140 saturation binding assay using HeLa cells with each point representing the average of duplicates. B_t_ = Btotal; Bns = Bnon-specific (determined in the presence of 10^−6^ M unlabeled T-140 peptide); Bsp = Bspecific (Bt-Bns).

6-[^18^F]FPy-T140 exhibited high-affinity binding with a K_d_ of 0.19 ± 0.03 nM (mean ± SE; *n* = 6) determined from saturation binding studies using the HeLa (moderate CXCR4 expression) cancer cell line (Figure 5). This K_d_ value compared favorably with previously reported IC_50_ values of the T-140 peptide (2.4 nM) and other T140 analogs (4-FBz-T140 analog = 0.99 nM, 1.75 nM; 4-F-T140 = 2.5 nM; T140-2D = 2.47 nM; Ac-Tz14011 = 1.2 nM; In-DPTA-Ac-TZ14011 = 7.9 nM; Ga-DOTA 4-FBz-TN14003 = 1.04 nM; DOTA-NFB T140 = 68 nM). The K_d_ value was at least 3-fold lower suggesting that this analog, 6-[^18^F]FPy-T140, has a higher affinity [24,31,48,49,50,51]. From the cell assays, the CXCR4 concentration (B_max_) on HeLa cells was 2.76 ± 0.51 × 10^5^ receptors per cell (mean ± SE; *n* = 6), which was consistent with immunofluorescence staining results by Peng et al. in which significant CXCR4 expression was observed with HeLa cells [52].

## 3. Materials and Methods

Non-radioactive standard (6-FPy-T140) and 1-(4,4-dimethyl-2,6-dioxocyclohexylidene) ethyl (Dde, **5**) protected peptide precursor T140 were obtained from CS Bio Co. (Menlo Park, CA, USA). Fluorine-18 was received from the National Institutes of Health cyclotron facility (Bethesda, MD, USA). Phosphate-buffered saline (PBS) 1X (12 mM phosphate buffer, pH 7.4, 137 mM NaCl, and 2.7 mM KCl) was obtained from Life Technologies (Carlsbad, CA, USA). All other chemicals and solvents were received from Sigma Aldrich (St. Louis, MO, USA) and used without further purification. For all fluorine-18 elutions, anhydrous solvents were used. Chromafix 30-PS-HCO_3_ anion-exchange Sep-Pak cartridges were purchased from Macherey-Nagel (Düren, Germany) and the packing material was reduced to half (~20 mg). Other columns and the Sep-Pak^®^ cartridges used in this synthesis were obtained from Agilent Technologies (Santa Clara, CA, USA) and Waters (Milford, MA, USA), respectively. Oasis MCX Plus cartridges were conditioned by passing 5 mL acetonitrile through them. Sep-Pak light C18 cartridges were conditioned with a sequence of 5 mL ethanol, 10 mL air, and 10 mL water. Mass spectra (MS) were recorded on a 6130 Quadrupole LC/MS, Agilent Technologies instrument equipped with a diode array detector. ^1^H, ^13^C, and ^19^F-NMR spectra were recorded on a 400 MHz Bruker spectrometer. Chemical shifts (ppm) were reported relative to the solvent residual peaks of dimethyl sulfoxide (δ ^1^H, 2.54 ppm; ^13^C 40.45), and chloroform (δ ^1^H, 7.26 ppm). ^19^F-NMR spectra were reported using trifluoroacetic acid as a reference (δ ^19^F, −76.72 ppm). High-performance liquid chromatography (HPLC) purification and analytical HPLC analyses for radiochemical work were performed on an Agilent 1200 Series instrument equipped with multi-wavelength detectors along with a flow count radio detector (Eckert & Ziegler, B-FC-3500 diode).

Method A (HPLC conditions for purification): Vydac C-4 (2) column (10 × 250 mm, 5 µ), mobile phase: 18% B in A; B = acetonitrile (0.1% TFA), A = Water (0.1% TFA), flow rate of 4 mL/min; t*_R_* = ~16 min.

Method B (HPLC conditions for analysis): Vydac C-4 (2) column (4.6 × 150 mm, 5 µ), mobile phase: 15–22% B in 10 min; B = acetonitrile (0.1% TFA), A = Water (0.1% TFA), flow rate of 1 mL/min; t*_R_* = ~8 min.

### 3.1. Synthesis of Precursor

#### Synthesis of 6-*N*,*N*,*N*-Trimethylammonium Nicotinic Acid NHS Ester Triflate Salt (**3**)

To a solution of 6-chloronicotinic acid NHS ester (**1**, 500 mg, 1.97 mmol) in anhydrous tetrahydrofuran (THF, 30 mL) at 0 °C, was added a solution of triethylamine (2 mL 1 M in THF, 2 mmol) with stirring. The reaction mixture was slowly allowed to warm up to room temperature and stirred for 24 h. The white precipitate was collected by filtration and washed with diethyl ether to produce the chloride salt of trimethylammonium nicotinic acid NHS ester (**2**, 500 mg, 1.6 mmol). To the suspension of **2** in anhydrous dichloromethane was added 1 mL trimethylsilyl trifluoromethanesulfonate (TMSOTf) and the mixture was stirred for 4 h followed by evaporation of the solvent under reduced pressure. The product was recrystallized by layering diethyl ether on top of the acetonitrile solution of **3**. After 4 days, the white crystals were collected and dried under reduced pressure (480 mg, 1.2 mmol, 55%, overall). ^1^H-NMR (400 MHz, DMSO-d6) δ 9.32 (d, *J* = 2.5 Hz, 1H), 8.92 (dd, *J* = 8.8, 2.4 Hz, 1H), 8.36 (d, *J* = 8.7 Hz, 1H), 3.65 (s, 9H), 2.94 (s, 4H). ^13^C-NMR (101 MHz, DMSO-d6) δ 170.4, 161.2, 160.20, 150.4, 143.4, 123.7, 117.1, 55.2, 26.1. ^19^F-NMR (376 MHz, DMSO-d6) δ -77.77. MS (Electrospray ionization, ESI) calculated mass for the parent C_13_H_16_N_3_O_4_ [M-OTf], 278.1, found 278.1 [M-OTf].

### 3.2. Synthesis of Side Product, 2,5-Dioxopyrrolidin-1-yl 6-((2,5-Dioxopyrrolidin-1-yl)Oxy)Nicotinate, *(**4**)*

To the solution of NHS (25.4 mg, 0.22 mmol) and triethylamine (5 µL) in anhydrous acetonitrile (1 mL) was added a solution of **3** (100 mg, 0.23 mmol) in anhydrous acetonitrile (1ml) and stirred for 1 h. The solvent was evaporated under reduced pressure. The residue was dissolved in dichloromethane and washed with water. The dichloromethane layer was dried with MgSO_4_ and the solvent was evaporated under reduced pressure to obtain **4** (60 mg, 0.18 mmol, 78%) as a white solid. ^1^H-NMR (400 MHz, Chloroform-d) δ 8.86 (s, 1H), 8.44 (d, *J* = 8.7 Hz, 1H), 7.22 (d, *J* = 8.7 Hz, 1H), 2.92 (d, *J* = 8.8 Hz, 8H). ^13^C-NMR (101 MHz, CDCl_3_) δ 169.4, 168.8, 164.2, 160.0, 150.7, 141.8, 119.2, 109.3, 25.6, 25.6. MS (ESI) calculated mass for the parent C_14_H_11_N_3_O_7_, 334.1 [M + H], found 334 [M + H]^+^.

### 3.3. Radiosynthesis of 6-[^18^F]FPy-T140

#### 3.3.1. Manual Synthesis

Fluorine-18 labeled target water (370–740 MBq) was diluted with 2 mL water and passed through an anion-exchange cartridge (Chromafix 30-PS-HCO_3_). The cartridge was washed with anhydrous acetonitrile (6 mL) and dried for 1 min under vacuum. The [^18^F]fluoride from the Sep-Pak was slowly eluted (0.5 mL/min) with 6-*N*,*N*,*N*-trimethylaminium nicotinic acid NHS ester triflate salt (**3**, 5–7 mg) in 0.5 mL 1:4 acetonitrile:*t*-butanol through an activated Oasis MCX Plus cartridge. The Sep-Pak was further eluted with 1 mL acetonitrile and the eluent was collected in the same vial. The solvent was evaporated under N_2_/vacuum at 45 °C. To the dried 6-[^18^F]SFP, was added a mixture of peptide precursor (**5**, 3–5 mg) in dimethyl sulfoxide (DMSO, 0.3 mL) and triethylamine (5 µL) followed by heating at 60 °C for 25 min. Hydrazine solution (0.5 mL of 2% solution, *v*/*v*) was added and kept at 60 °C for 10 min. HPLC buffer (18% acetonitrile in water with 0.1% TFA; 3 mL) was added and the mixture was injected into the HPLC for purification (method A). 

#### 3.3.2. Automated Synthesis

Fluorine-18 labeled target water (3700–7400 MBq) was diluted with 2 mL water and passed through an anion-exchange cartridge (Chromafix 30-PS-HCO_3_) followed by anhydrous acetonitrile (6 mL) and the cartridge was dried for 1 min under vacuum. The [^18^F]fluoride from the Sep-Pak was eluted with either the NHS ester (3) or TFP ester of the triflate salt of 6-*N*,*N*,*N*-trimethylaminium nicotinic acid (5–7 mg) in 0.5 mL 1:4, acetonitrile:t-butanol (in a syringe) via the external three-way valve. The mixture was passed through a pre-conditioned Oasis MCX Plus cartridge (incorporated between V13 and Reactor 1). The cartridge was flushed with 1 mL acetonitrile using the same line and the eluent was collected in the Reactor 1. The solvent was evaporated under N_2_/vacuum either at 45 °C. To the dried fluorine-18 labeled reactive ester in Reactor 1 was added a mixture of peptide precursor (**5**, 3–5 mg) and triethylamine (5 µL) in DMSO (0.5 mL) from Vial 3. The solution was stirred for 25 min at 60 °C followed by the addition of hydrazine solution (0.5 mL 2%, *v*/*v*) from Vial 4 and stirring was continued for 10 min at 60 °C. HPLC buffer (3 mL) was added from Vial 5 and the solution was transferred to Tube 2 for HPLC purification. The product fraction was collected in the dilution flask containing 20 mL water. The solution was passed through a Sep-Pak light C18 cartridge to retain the product. The cartridge was washed with water (6 mL) from Vial 14. The product was eluted with ethanol (1 mL) from vial 13 followed by PBS 1X (3 mL) from Vial 12 in a product vial.

### 3.4. In Vitro Studies

The human cervical carcinoma cell line, HeLa (moderate CXCR4 expression), was purchased from ATCC (Gaithersburg, MD) and grown in DMEM supplemented with 4 mM L-glutamine, 1 mM sodium pyruvate, 10% fetal bovine serum (FBS) and 1% Pen/Strep/Amphotericin B at 37 °C in a 5% CO_2_ humidified atmosphere. 

For the saturation assays sub-confluent HeLa cells were harvested (0.125% Trypsin-EDTA) and resuspended in binding buffer (RPMI 1640 containing 20 mM HEPES and 0.5% (*w*/*v*) bovine serum albumin (BSA), pH 7.0).

Saturation studies were performed by adding increasing concentrations of 6-[^18^F]FPy-CONH-T140 (0.05 nM to 2.5 nM; B_t_)) to duplicate tubes with a constant concentration of HeLa cells (0.50 to 1.1 × 10^5^ cells per tube); non-specific binding (B_nsb_) was determined by adding unlabeled 6-FPy-T140 peptide (10^−6^ M) to another set of duplicates at the same labeled peptide concentrations. Following incubation for 1 h at 37 °C, the cell-bound 6-[^18^F]FPy-T140 was separated from the free radioligand by centrifuging, and washing twice (PBS). After aspirating the samples, the radioactive content of the cell pellets was determined by gamma counting (Perkin Elmer 2480 Wizard 2, Shelton, CT). The K_d_ and B_max_ were determined from 6 to 8 concentrations of 6-[^18^F]FPy-T140 and analyzed using non-linear regression curve fits including a one-site binding hyperbola for saturation studies (GraphPad PRISM version 7.05 for Windows, GraphPad Software, San Diego, CA, USA; www.graphpad.com). 

## 4. Conclusions

6-[^18^F]SFPy was prepared in high yield using ‘fluorination on the Sep-Pak’ method. The conjugation of 6-[^18^F]SFPy with peptide precursor was used to produce a new tracer, 6-[^18^F]FPy-T140. The simplicity of this labeling procedure allows us to successfully develop an automated synthesis method. In vitro assays demonstrated that 6-[^18^F]FPy-T140 exhibited sub-nanomolar binding affinity (Kd ~0.2 nM) for CXCR4 indicating that the biological activity of the peptide had been retained.
